# The Effect of Acupuncture on Elbow Joint Sports Injuries Based on Magnetic Resonance Imaging

**DOI:** 10.1155/2022/9005792

**Published:** 2022-04-04

**Authors:** Weihua Yang, Fei Wang

**Affiliations:** ^1^Xinxiang University, Xinxiang Henan 453003, China; ^2^Hainan General Hospital, Haikou Hainan 570311, China

## Abstract

**Purpose:**

Elbow joint injuries are extremely common in most athletes. Athletes' chronic elbow injuries can involve multiple complex anatomical structures related to orthopedics. Therefore, it is of great significance to explore the characteristics of magnetic resonance (MR) images of chronic sports injuries of the elbow joint and the influence of acupuncture treatment on MR images and clinical symptoms.

**Methods:**

A total of 60 elbow joints of 39 athletes from 15-25 years old were selected for coronal, sagittal, and axial MR scans to observe the image characteristics; 60 elbow joints were randomly divided into acupuncture group and control group and observed changes in MR images and clinical symptoms after acupuncture treatment.

**Results:**

After acupuncture treatment, the clinical symptoms were significantly improved. MR images showed that the elbow joint effusion was reduced, and the bone marrow edema was reduced. The effective rate of acupuncture treatment is as high as 100%, while the effective rate of the control group is only 40%. Acupuncture treatment has significantly improved the range of motion of the elbow joint.

**Conclusion:**

Acupuncture treatment can significantly relieve athletes' elbow joint pain and locking symptoms, improve joint range of motion, and is beneficial to recovery of special training and high-level competitive competitions. It is a sensitive, effective, and noninvasive method.

## 1. Introduction

With the continuous development of modern competitive gymnastics, athletes' injuries also occur frequently, which seriously affects the normal systematic training of athletes. Competitive gymnasts' injuries mostly occur in the wrist, ankle, and elbow joints, and the ankle joint has the highest injury rate. The elbow joint is one of the most important joints in the human body, and it is also the joint that experiences the greatest load, the most unstable and easy to be injured in the whole body joints [1]. Therefore, this paper analyzes and discusses the mechanism, prevention, treatment, and rehabilitation training of elbow joint injury and aims to provide theoretical basis for the teaching and training of competitive gymnastics.

Early diagnosis of sports injuries and how to extend the sports life span of athletes are the most fundamental tasks of sports medicine. The occurrence of sports injury diseases is not only different in pathogenesis but also in athletes' treatment and prognosis [2, 3]. Elbow injuries are extremely common among athletes in gymnastics, tennis, judo, weightlifting, badminton, javelin, skills, basketball, table tennis, and other sports. Athletes' chronic elbow injuries can involve multiple epiphyses, cartilage, synovium, ligaments, soft tissues, and other complex anatomy.

The elbow joint is the main weight-bearing joint of the human body and one of the vulnerable joints in daily life and sports competitions [4, 5], accounting for about 40% of all sports injuries. The ligaments around the elbow joint play an important role in maintaining the stability of the elbow joint. Its injuries are often complicated by elbow joint fractures, and other normal anatomical structures around the joint are destroyed and joint instability. If not treated properly, patients are prone to joint laxity and subsequent cartilage injury, traumatic osteoarthritis, and so on [6, 7]. Accurately determining the damage to the ligaments around the elbow joint is very important for the treatment and prognosis of the patient.

The anatomical structure of the human elbow joint is complex. At present, the clinic mainly relies on routine X-ray examination of ankle injuries. With the development of clinical imaging technology, magnetic resonance imaging (MRI) has gradually been recognized and promoted [8–10]. Currently, the imaging technology has high resolution and noninvasiveness for soft tissues due to the characteristics of sex and nonradiation damage have been widely used in clinical examination of various system diseases of the human body [11]. Recently, clinical studies have found that the implementation of MRI diagnosis for patients with elbow joint ligament injury has a good diagnostic effect.

At present, conventional X-ray films have a clear diagnosis of fractures, but the evaluation of chronic elbow injuries in athletes is more limited, and certain types of epiphyseal injuries often cause difficulties in diagnosis and treatment [12–14]. MRI can clearly show cartilage, synovia, ligaments, soft tissues, and blood vessels. It is sensitive to show changes in bone marrow and can detect bone damage, osteomyelitis, aseptic necrosis, etc. early. Acupuncture treatment of elbow joint injury is mostly based on the local injury and combined with remote point differentiation.

## 2. Methods and Materials

### 2.1. Patient Demographics

This group of 39 athletes with chronic elbow joint sports injury has a total of 60 elbow joints, and there are 6 selection criteria: (1) Elbow pain, swelling, elbow joint dysfunction, or limited mobility; (2) X-ray films exclude pathological fractures; (3) criteria do not involve major nerve and blood vessel injury; (4) 15-25 years old; (5) uninterrupted treatment as prescribed; (6) complete follow-up obtained. Cases meeting the above inclusion criteria are eligible cases.

**Figure 1:**
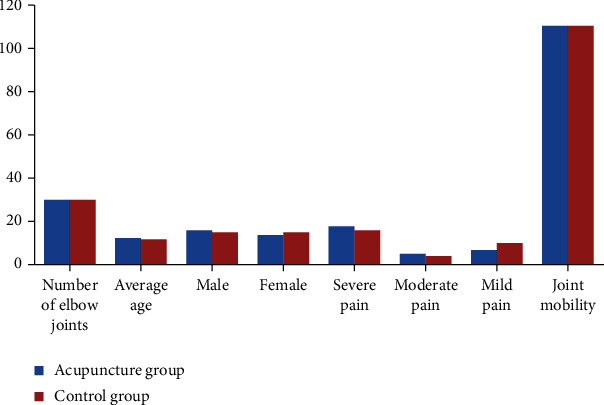
Comparison of symptoms and signs between the two groups.

Among them are 36 right elbows and 24 left elbows; 31 males and 29 females (19.3 ± 6.1) years old; all are professional athletes, including 22 elbows for gymnastics, 15 elbows for tennis, 4 for weightlifting, and 4 for badminton. 3 elbow joints for javelin, judo, technique, basketball, and table tennis individually; course of disease (12.5 ± 24.8) months; follow-up time (12.2 ± 14.5) months. The 60 elbow joints were randomly divided into acupuncture group and control group according to age, gender, pain degree, and joint range of motion according to the random number table method. The acupuncture group had 30 elbow joints, and the control group had 30 elbow joints. There were 2 groups of symptoms, the signs and conditions were not significantly different after analysis, and they were comparable (*P* > 0.05), as shown in Figure 1.

### 2.2. Treatment Methods

Acupuncture is used in this study, and the skin is routinely disinfected by acupuncture points. When acupuncture Chize point, we use a 1.5-inch needle with the tip of the needle at an angle of 30° to the skin and quickly penetrate into the skin. Stop when there is a feeling of tightness and stagnation under the fingers. Insert the needle and then twist the needle body at a speed of 200 revolutions/min for 2 minutes; retract the needle to the subcutaneous position, and insert the needle tip to the left at an angle of 90° to the front and back midline of the elbow. We repeat the above operations.

Needle is penetrated backward and rightward, and the operation is the same as before. When acupuncture other points, quickly pierce the needle under the skin, and then slowly insert the needle with a twisting technique. When there is a feeling of tightness and stagnation under the fingers, stop the needle and insert the needle with the twisting technique for 2 minutes. We keep the needle for 30-60 minutes and row the needle every 15 minutes. Once a day, 15 times are a course of treatment. The interval between each treatment is 2 d. There are 4 courses of treatment. Control group: stop training with affected elbow and rest.

### 2.3. MR Scanning Method

All subjects used GE HD Excite 1.5 T MRI instrument to perform conventional sagittal, coronal, and axial multisequence, multiparameter scans, sagittal FRFSE T2WI fs (TR2000ms, TE60-80 ms), FSET1WI (TR500ms, TE20ms), STIR T2∗WI (TR2000ms, TE30ms), coronal FSE T1WI, PDWI (TR2000ms, TE20-30 ms), and axial FSE T1WI (TR1000ms, TE40ms).

### 2.4. Evaluation Procedures

After 4 courses of treatment, the two groups were uniformly evaluated for curative effect. The modified Hospital for Special Surgery (HSS) elbow score was used as the evaluation criterion for efficacy. (1) Cured: the local redness, swelling, and pain disappeared, and the range of motion of the joint returned to normal (the range of motion of the elbow joint was ≥110°); (2) significant effect: the local redness, swelling, and pain basically disappeared, there was no obvious restriction on activities of daily living, and the range of motion of the elbow joint was 90-110°; (3) improved: local swelling and pain are relieved, activities of daily living are mildly limited, and the range of motion of joints is 60-90°; (4) ineffective: local swelling and pain are not significantly improved, and activities of daily living are significantly improved limited, joint range of motion <60°. The overall efficacy includes cure, marked improvement, and improvement.

### 2.5. Statistical Analysis

We analyzed the data with SPSS11.0 statistical software and performed *χ*^2^ test on the data.

## 3. Results

### 3.1. Comparison of MR Images Pre- and Postacupuncture Treatment

**Figure 2:**
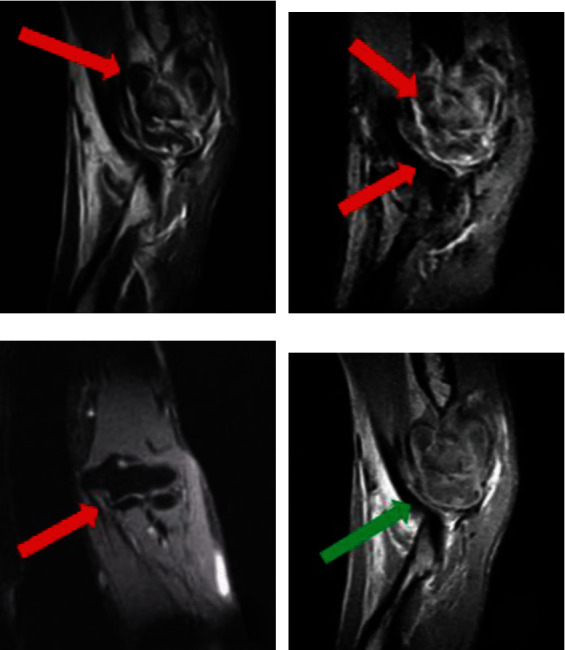
MR images before and after acupuncture treatment based on (a) MR image of elbow joint injury feature one before treatment, (b) MR image of elbow joint injury feature two before treatment, (c) MR image of elbow joint injury feature three before treatment, and (d) MR image after acupuncture treatment. The red arrow represents the injury site before treatment, and the green arrow represents the recovery position after treatment.

The first feature is the thickening of the elbow joint capsule ligament and synovial membrane, the joint space of the inner elbow can be seen larger than normal; the synovial membrane can also be seen unevenly thickened, the PDWI sequence shows high signal, synovial effusion, manifested as strip hypo-intensity signal on T1WI, and hyperintensity signal on T2WI. The specific image is shown in Figure 2(a).

The second feature is that the ulnar collateral ligament is interrupted by normal low signal in PDWI sequence. A few athletes can see low signal calcification of muscles and tendons. The ossification or calcification and the surrounding soft tissue scars, inflammation, contracture, and incarceration can all cause elbow mobility disorder. The surrounding soft tissues are swollen, and uneven hyperintensity signals can be seen in the soft tissues on both T2WI and PDWI sequences. It is more common in elbow-ulnar pain syndrome, as shown in Figure 2(b).

The third feature is the cartilage injury of the capillary humerus and the internal epicondylitis of the humerus. The manifestations are the internal epicondyle of the humerus and the articular surface of the humerus. The cartilage signal is defective. And on the PDWI sequence, hyperintensity signals can be seen in the capillary cartilage of the humerus, the olecranon, and the coronoid process of the ulna, as shown in Figure 2(c).

After acupuncture treatment, MR images showed that the elbow joint effusion was reduced, the joint cavity effusion was reduced, the synovial sac was not significantly thickened, and the bone marrow edema was reduced, as shown in Figure 2(d).

### 3.2. Comparison of Clinical Manifestations before and after Treatment

Before treatment, 60 elbow joints had pain, of which 34 were obvious pain, 9 were moderate pain, and 17 were mild pain. The specific elbow joint training degree and bending degree are shown in Table 1.

**Figure 3:**
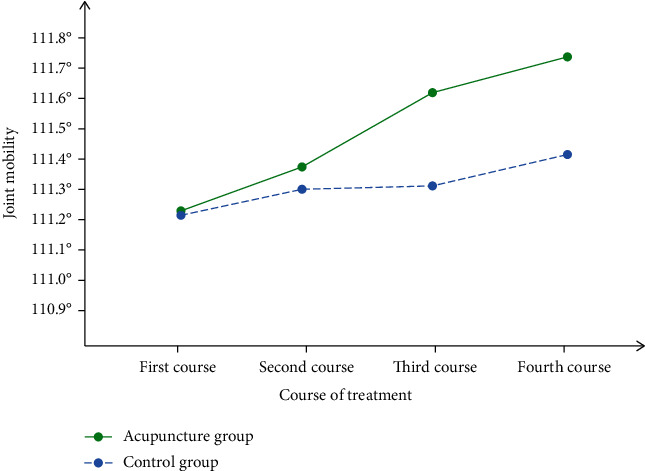
Changes in elbow joint range of motion in four courses.

Acupuncture treatment is divided into 4 courses. In order to better compare the effects of acupuncture treatment, we carefully recorded the changes in elbow joint mobility during the 4 courses, as shown in Figure 3.

It can be seen from Figure 3 that the elbow joint range of motion in the control group did not change much, while the acupuncture group increased the range of motion after 4 courses of treatment. Although it only increased by 0.5°, the range of motion has also increased a lot. It also affects the daily efficiency of athletes.

The follow-up time after acupuncture treatment was (12.2 ± 14.5) months. Among the 30 elbows in the acupuncture group, 21 elbow pain disappeared, 6 elbow joint pain basically disappeared, activities of daily living were not significantly restricted, the pain degree of 3 elbow joints was reduced, and the apparent rate was 100%. Of the 30 elbows in the control group, 4 elbow pain disappeared, 3 elbow joint pain basically disappeared, there was no obvious restriction in activities of daily living, 5 elbow joint pain was reduced, 18 elbow joint pain was not improved significantly, daily life activity capacity is obviously limited, and the apparent efficiency is 40%. The treatment efficiency of the acupuncture group and the treatment group is shown in Table 2.

**Figure 4:**
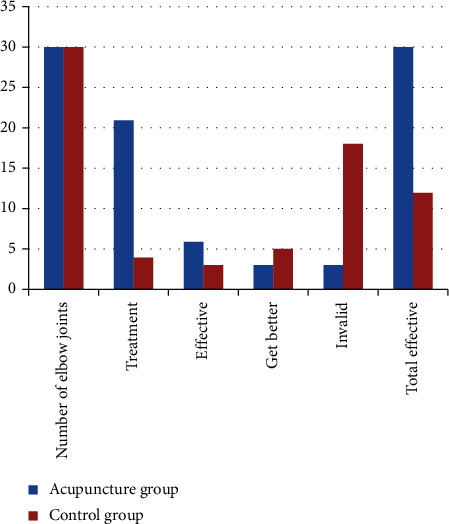
Comparison of elbow joint treatment effects between the two groups.

Figure 4 shows the comparison of elbow joint treatment effects between the two groups, showing the specific number of elbow joints. We can easily conclude that the treatment effect of the acupuncture group is better than that of the control group.

## 4. Discussion

When the extension, flexion, and rotation of the elbow joint exceed a certain range of angles, or when the joint cannot perform any movement, it can cause injury. All joint surfaces are covered by articular hyaline cartilage. To evaluate articular cartilage, it is best to use GRE sequence or fat-saturated proton density-weighted image [15], while X-ray and CT cannot show articular hyaline cartilage. The medial collateral ligament of the elbow joint plays an important role in the movement of the elbow joint. Chronic elbow joint instability [16] mainly involves chronic injury of the elbow collateral ligament. MR can show the anatomy of the elbow collateral ligament and clearly distinguish the anterior medial collateral ligament. The anterior and posterior tracts of the medial collateral ligament at different flexion angles have been found to play a role, respectively, in the study [17].

Due to the abnormal range of the elbow joint's out-of-groove motion, excessive extension, flexion, twisting, and supporting load, the cartilage surface of the elbow joint is constantly squeezed, rubbed, and collided and gradually strained and injured. At the same time, traumatic osteochondritis can lead to secondary elbow joint osteoarthropathy [18]. After the primary injury is healed, hematomas in the joint cavity gradually become organized, and the joint capsules and soft tissues around the joints such as muscles, tendons, ligaments, and other soft tissues contracture adhesion [19], there are joint extension and flexion disorders, joint swelling, and muscle atrophy.

Acupuncture treatment is based on different diseases and syndromes, and the treatment is based on syndrome differentiation, so that the local blood vessels are unobstructed, the muscles and bones are nourished, and the joints are flexible in flexion and extension. The treatment of this disease is based on the principles of dredging the meridians, relieving spasms, loosening adhesions, improving blood circulation in local tissues, relieving pain, and promoting joint function to normal [20]. Acupuncture treatment achieves the purpose of smoothing joints, relieving adhesions, improving joint function, and eliminating inflammation and edema [21, 22].

Although the acupuncture treatment method proposed in this paper has achieved a significant efficiency of 100%, there are still many problems and areas that can be improved [23].

First, we should expand the experimental cases. This paper only uses 39 cases of elbow joint injuries, but to draw more accurate conclusions, we need more cases to further prove our point [24].

Second, in MR images, three-dimensional factors are added [25]. The images in this paper are all two-dimensional, and there is a risk of ignoring important details of the elbow joint injury. Deep learning can be used for automatic diagnosis of the patient in future [26–29].

Third, we will study more common elbow injuries in the future. This paper mainly studies athletes' elbow joint injuries, but in reality, the situation of ordinary people's elbow joint injuries is more complicated [30], and future research directions will work hard in this area.

## 5. Conclusion

The HSS scoring method was used before and after treatment in all cases. Pain and functions of daily living accounted for a significant proportion of the scores, indicating that attention should be paid to improving the overall function of the elbow joint and improving the activities of daily living of the affected limb. After more than 3 months of follow-up, the apparent efficiency of the acupuncture group reached 100%, indicating that the acupuncture treatment can restore joint flexion to the greatest extent.

In the examination of athletes' elbow injuries, especially those involving complex elbow joint injuries, MR examination can take full advantage of its noninvasive, reproducible, and high-resolution advantages of soft tissue structure, combined with multiple scan orientations, multiple scan parameters, and pass continuous. Scanning observation can make a comprehensive judgment. Not only that, MR examination can play a role in guiding treatment by evaluating the stability of trauma fractures and the stability after treatment. In addition, it is generally believed that MR examination can detect cartilage callus formation earlier than plain X-ray films, and it can also be used to monitor the healing of fractures.

## Figures and Tables

**Table 1:** The degree of elbow joint training and the degree of bending before treatment.

Group	Trauma	No trauma	Participate in normal special training			Angle of elbow extension	The angle of the elbow joint
			Cannot	Impact	Obvious impact		
Acupuncture group	14	16	7	10	13	14.9° ± 13.4°	119.9° ± 13.4°
Control group	9	21	10	6	14	15.1° ± 12.7°	118.8° ± 9.9°

**Table 2:** The treatment efficiency of acupuncture group and treatment group.

Group	Number of elbow joints	Treatment (%)	Effective (%)	Get better (%)	Invalid (%)	Total effective rate (%)
Acupuncture group	30	70.00	20.00	10.00	0.00	100.00
Control group	30	13.33	10.00	16.67	60	40.00

## Data Availability

The image data used to support the findings of this study have been deposited in the musculoskeletal radiographs (MURA) dataset (https://stanfordmlgroup.github.io/competitions/mura/).
